# Crystal structures of two ytterbium(III) complexes comprising alkynylamidinate ligands

**DOI:** 10.1107/S2056989016012135

**Published:** 2016-08-02

**Authors:** Sida Wang, Farid M. Sroor, Phil Liebing, Volker Lorenz, Liane Hilfert, Frank T. Edelmann

**Affiliations:** a Chemisches Institut der Otto-von-Guericke-Universitaet Magdeburg, Universitaetsplatz 2, 39106 Magdeburg, Germany; b Organometallic and Organometalloid Chemistry Department, National Research, Centre, 12622 Cairo, Egypt

**Keywords:** crystal structure, ytterbium, amidinates, alkynylamidinate ligands, cyclo­penta­dienyl complexes

## Abstract

Two ytterbium(III) complexes comprising alkynylamidinate ligands, namely bis­(η^5^-cyclo­penta­dien­yl)(3-cyclo­propyl-*N*,*N*′-diiso­propyl­propynamidinato-κ^2^ *N*,*N*′)ytterbium(III), [Yb(C_5_H_5_)_2_(C_12_H_19_N_2_)] or Cp_2_Yb[(^*i*^Pr_2_N)_2_C—C≡C—*c*-C_3_H_5_] (**1**) and tris­(3-phenyl-*N*,*N*′-di­cyclo­hexyl­propynamidinato-κ^2^ *N*,*N*′)ytterbium(III), [Yb(C_21_H_27_N_2_)_3_] or Yb[(CyN)_2_C—C≡C—Ph]_3_ (Cy = cyclo­hex­yl) (**2**) have been synthesized and structurally characterized. Both complexes are monomers; for complex **2**, the contribution to the scattering from highly disordered toluene solvent molecules in these voids was removed with the SQUEEZE routine [Spek (2015). *Acta Cryst.* C**71**, 9–18] in *PLATON*. The stated crystal data for *Mr*, μ *etc*. do not take these into account.

## Abstract 2

Two ytterbium(III) complexes comprising alkynylamidinate ligands, Cp_2_Yb[(^*i*^Pr_2_N)_2_C—C≡C—*c*-C_3_H_5_] (**1**) and Yb[(CyN)_2_C—C≡C—Ph]_3_ (Cy = cyclo­hex­yl) (**2**) have been synthesized and structurally characterized. Both complexes are monomers without any coordinated solvent in the solid state.

## Chemical context   

Anionic amidinate ligands of the type [*R*C(N*R*′)_2_]^−^ (*R* = H, alkyl, aryl; *R*′ = alkyl, cyclo­alkyl, aryl, SiMe_3_) are highly useful and versatile spectator ligands in organolanthanide chemistry. These readily available *N*-chelating ligands are generally regarded as sterically demanding cyclo­penta­dienyl equivalents (Collins, 2011 ▸; Edelmann, 2013 ▸). Mono-, di- and tris­ubstituted lanthanide amidinate complexes are all accessible, in close analogy to the long known mono-, di- and tri­cyclo­penta­dienyl complexes. Over the past *ca* 25 years, lanthanide amidinates have witnessed an impressive transformation from laboratory curiosities to homogeneous catalysts as well as valuable precursors in materials science. Rare-earth metal amidinates have been reported to be highly active homogeneous catalysts *e.g.* for ring-opening polymerization reactions of lactones, the guanylation of amines or the addition of terminal alkynes to carbodi­imides (Edelmann, 2009 ▸, 2012 ▸). In materials science, certain homoleptic alkyl-substituted lanthanide tris(amidinate) complexes are highly volatile and can be used as precursors for ALD (atomic layer deposition) and MOCVD (metal–organic chemical vapor deposition) processes, *e.g.* for the deposition of lanthanide oxide (*Ln*_2_O_3_) or lanthanide nitride (*Ln*N) thin films (Devi, 2013 ▸).

Introduction of alkynyl groups to the central C atom in amidines provides alkynyl­amidines of the general type *R*—C≡C—C(N*R*′)(NH*R*′). In organic synthesis, alkynyl­amidines have been frequently employed in the preparation of various heterocycles (Ong *et al.*, 2006 ▸; Xu *et al.*, 2008 ▸; Weingärtner & Maas, 2012 ▸). Alkynyl­amidines are also useful for diverse applications in biological and pharmacological systems (Rowley *et al.*, 2005 ▸; Sienkiewicz *et al.*, 2005 ▸). Thus far, only a few lanthanide complexes containing alkynylamidinate ligands have been described. Previously used alkynylamidinate ligands include *e.g.* phenyl­ethynyl derivatives [Ph—C≡C—C(N*R*)_2_]^−^ (*R* = ^*i*^Pr, ^*t*^Bu) (Dröse *et al.*, 2010*a* ▸,*b* ▸; Xu *et al.*, 2013 ▸) and the tri­methyl­silyl-substituted anions [Me_3_Si—C≡C—C(N*R*)_2_]^−^ [*R* = cyclo­hexyl (Cy), ^*i*^Pr] (Seidel *et al.*, 2012 ▸). 
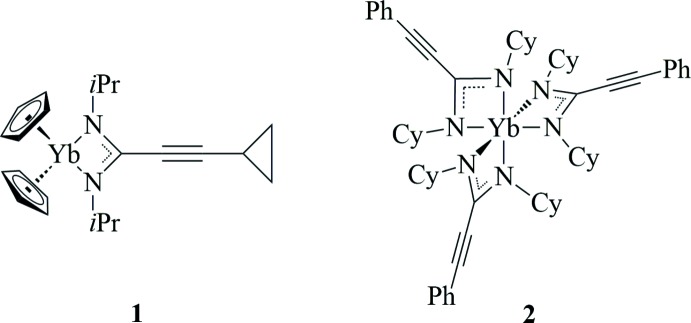


**Figure 1:**
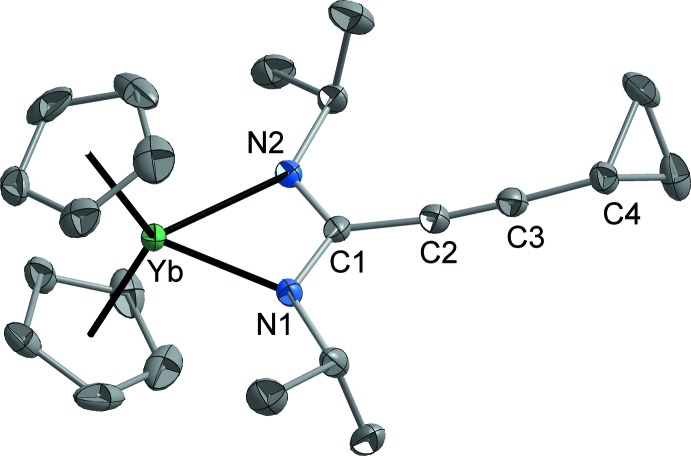
The mol­ecular structure of compound **1**.

We recently initiated a study of alkynylamidinates derived from cyclo­propyl­acetyl­ene (Sroor *et al.*, 2015*c* ▸). The cyclo­propyl group was chosen because of the well-known electron-donating ability of this substituent to an adjacent electron-deficient atom or group. This would give us the rare chance to electronically influence the amidinate ligand system rather than altering only its steric demand. We now describe the synthesis and structural characterization of two new ytterbium(III) alkynylamidinate complexes, namely Cp_2_Yb[(^*i*^PrN)_2_C—C≡C—*c*-C_3_H_5_] (**1**) and Yb[(CyN)_2_C—C≡C—Ph]_3_ (Cy = cyclo­hexyl; **2**), shown in Figs. 1 ▸ and 2 ▸.

## Structural commentary   

The structural analyses revealed that both title compounds are monomeric in the solid state, with the alkynylamidinate anion acting as an *N*,*N*′-chelating ligand. Compound **1** crystallizes in the ortho­rhom­bic space group *Pbca* with one complex mol­ecule in the asymmetric unit. The two cyclo­penta­dienyl ligands feature a typical symmetric η^5^-coordination with Yb–centroid(Cp) distances of 2.315 and 2.321 Å. The Yb—Cp distances are therefore slightly larger than in the related chloride [Cp_2_YbCl]_2_ [Yb–centroid(Cp) 2.300 and 2.307 Å; Lamberts *et al.*, 1987 ▸; Lueken *et al.*, 1987 ▸, 1989 ▸], possibly due to the steric demand of the two *N*-isopropyl groups close to the ytterbium atom. Probably for the same reason, the product does not contain coordinating THF even though the complex was prepared in THF solution. Accordingly the coordination geometry around Yb can be described as distorted pseudo-tetra­hedral. At 131.1°, the Cp—Yb—Cp angle is close to that observed in [Cp_2_YbCl]_2_ (Cp—Yb—Cp 130.0°; Lamberts *et al.*, 1987 ▸; Lueken *et al.*, 1987 ▸, 1989 ▸) and compound **1** is therefore a typical bent metallocene complex of trivalent ytterbium. Due to the low formal coordination number of four around the Yb atom, the Yb—N bond lengths of 2.274 (2) and 2.293 (2) Å are short compared to those observed in other late lanthanide amidinates, such as [Yb_2_{(DippN)_2_CH}_4_(μ-OCPh=C_6_H_4_-4-CPh_2_O)(THF)] [Yb—N 2.285 (2)–2.391 (2) Å; Deacon *et al.*, 2014 ▸], [Ho{N(SiMe_3_)_2_}{(CyN)_2_C—C≡C—*c*-C_3_H_5_}_2_] [Ho—N 2.303 (2)–2.348 (4) Å; Sroor *et al.*, 2015*b* ▸] and [Ho(η^8^-COT){(CyN)_2_C—C≡C—*c*-C_3_H_5_}(THF)] [Ho—N 2.342 (3) and 2.349 (3) Å; Sroor *et al.*, 2016 ▸].

Compound **2** crystallizes in the trigonal space group *R* 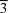 *c*, with the Yb atom located on a threefold rotation axis along the crystallographic *c* axis. The complex mol­ecule is therefore *C*3 symmetric. The Yb atom is coordinated by the three symmetry-equivalent chelating amidinate ligands in a distorted octa­hedral fashion with C1—Yb—C1′ angles of 120° and an angle of 90±3° between the YbN_2_C planes. The cyclo­hexyl group attached to N2 is disordered over two orientations by rotation around the N2—C16 vector. As a result of the higher coordination number, the Yb—N bonds [2.310 (2) and 2.320 (2) Å] are slightly longer than in compound **1**. However, in consequence of the small size of the Yb^3+^ ion, the Yb—N bonds in compound **2** are significantly shorter than in corres­ponding hexa­coordinated lanthanide(III) amidinates, *e.g.* [*Ln*{(^*i*^PrN)_2_C–^*t*^Bu}_3_] [*Ln* = Ce: Ce—N 2.469 (2)–2.550 (2) Å; *Ln* = Eu: Eu—N 2.402 (4)–2.457 (4) Å; *Ln* = Tb: Tb—N 2.391 (3)–2.409 (3) Å; Dröse *et al.*, 2011 ▸] and [Ho{(CyN)_2_C—C≡C—*c*-C_3_H_5_}_3_] [Ho—N 2.342 (2)–2.383 (3) Å] (Sroor *et al.*, 2015*a* ▸).

The N—Yb—N angle in compound **2** [58.2 (1)°] is slightly smaller than in compound **1** [59.1 (1)°], but larger than in other homoleptic lanthanide (III) amidinates {*e.g.* [*Ln*{(^*i*^PrN)_2_C–^*t*^Bu}_3_], *Ln* = Ce: N—Ce—N 51.81 (4)–52.72 (4)°; *Ln* = Eu: N—Eu—N 53.9 (1)–54.4 (2)°; *Ln* = Tb: N—Tb—N 54.9 (1)–55.0 (1)°; Dröse *et al.*, 2011 ▸} and [Ho{(CyN)_2_C—C≡C—*c*-C_3_H_5_}_3_] [N—Ho—N 57.1 (1)–57.7 (1)°; Sroor *et al.*, 2015*a* ▸]. The N—*Ln*—N angle therefore correlates clearly with the *Ln*—N bond length, decreasing with rising *Ln*—N distance (*i.e.* with rising coordination number of the metal and within the lanthanide series from right to left). The C1—N bond lengths of the amidinate ligand are very similar [**1**: 1.332 (3) and 1.334 (3) Å; **2**: 1.321 (4) and 1.324 (4) Å], indicating a typical delocalization of the negative charge within the NCN fragment (Sroor *et al.*, 2016 ▸).

## Supra­molecular features   

Compounds **1** and **2** do not exhibit any specific inter­molecular inter­actions. In compound **1**, the closest inter­molecular C—C contacts are found between Cp ligands and cyclo­propyl substituents, 3.510–3.625 Å. Compound **2** features one inter­molecular phen­yl–cyclo­hexyl contact where the shortest C—C distance is 3.567 Å, and various cyclo­hex­yl–cyclo­hexyl contacts with C—C distances of 3.441–3.576 Å. The crystal structure of compound **2** comprises a large void of *ca* 220 Å^3^ that is probably filled with a highly disordered toluene mol­ecule. The content of the voids was corrected for using the SQUEEZE method (Spek, 2015 ▸), yielding a solvent-accessible volume of 1316 Å^3^ and 138 electrons, or about 1.5 solvate mol­ecules per unit cell. The composition of the crystal can therefore be assumed to be **2**·0.166 toluene.

## Database survey   

For other lanthanide(III) complexes with amidinate ligands, see Richter *et al.* (2004 ▸), Edelmann (2009 ▸, 2012 ▸) and Deacon *et al.* (2014 ▸). For related bent sandwich complexes of the lanthanides, see Lueken *et al.* (1987 ▸, 1989 ▸), Schumann *et al.* (1998 ▸) and Kühling *et al.* (2015 ▸).

## Synthesis and crystallization   

**Synthesis of Cp_2_Yb[(** ***^i^*** **Pr_2_N)_2_C–C**≡**C–** ***c*** **-C_3_H_5_] (1)**

This compound was prepared by treatment of Cp_2_YbCl (Maginn *et al.*, 1963 ▸) with Li[(^*i*^Pr_2_N)_2_C—C≡C—*c*-C_3_H_5_] (Sroor *et al.*, 2013 ▸) in a molar ratio of 1:1. Treatment of Cp_2_YbCl (0.68 g, 2.0 mmol) with Li[(^*i*^Pr_2_N)_2_C—C≡C—*c*-C_3_H_5_] (2.0 mmol, prepared *in situ* from Li—C≡C—*c*-C_3_H_5_ and *N*,*N*′-diiso­propyl­carbodi­imide) in 30 ml of THF produced a bright-orange solution and a white precipitate (LiCl). After filtration and evaporation to dryness, the product was extracted with *n*-pentane (2 × 20 ml). The extract was filtered again and concentrated to a total volume of *ca* 10 ml. Crystallization at 253 K afforded **1** as orange air- and moisture-sensitive crystals. Yield: 0.53 g, 73%. M.p.: 478 K. Analysis calculated for C_22_H_20_N_2_Yb: C 53.43, H 5.91, N 5.66%; found: C 53.61, H 5.766, N 5.86%. MS (EI, *M* = 494.54): *m*/*z* (%) 450 (5) [*M* − 3CH_3_]^+^, 407 (5) [*M* − 2^*i*^Pr]^+^, 384 (7), 369 (13) [*M* − 2Cp + 3H]^+^, 355 (5), 341 (66) [*M* − Cp − 2^*i*^Pr]^+^, 328 (5), 313 (4) [YbN^*i*^Pr—C(CH)—N^*i*^Pr]^+^, 299 (7) [YbN^*i*^Pr—C—N^*i*^Pr]^+^, 284 (10) [YbN^*i*^Pr—C—NCCH_3_]^+^, 274 (100) [YbN^*i*^Pr—C—NCH_3_]^+^, 258 (25) [YbN^*i*^Pr—CN]^+^, 243 (8), 232 (10), 215 (12) [YbNCN]^+^. IR (KBr) ν (cm^−1^): 3093 (*w*), 2963 (*m*), 2922 (*w*), 2871 (*w*), 2609 (*w*), 2215 (*m*, C≡C), 2070 (*w*), 1985 (*w*), 1746 (*w*), 1609 (*m*, NCN), 1450 (*s*), 1367 (*m*), 1327 (*m*), 1258 (*w*), 1224 (*m*), 1177 (*m*), 1055 (*w*), 1012 (*m*), 968 (*m*), 878 (*w*), 766 (*vs*), 695 (*m*), 531 (*w*), 481 (*w*), 393 (*w*), 328 (*w*). ^1^H NMR (400 MHz, [D_6_]-benzene, 298 K): δ 0.92 (overlapped, *m*, C*H*-cyclo­prop­yl), 0.47–0.51 (*m*, 2H, C*H*_2_-cyclo­prop­yl), 0.25–0.20 (*m*, 2H, C*H*_2_-cyclo­prop­yl), −1.5 (*br s*, 10H, C*H* Cp), −7.2 (1H, sept, C*H ^i^*Pr), −10.8 (1H, sept, C*H ^i^*Pr), −36.9 (*br s*, C*H*_3_^*i*^Pr). ^13^C NMR (100.6 MHz, [D_6_]-benzene, 298 K): δ 152.3 (*s*, N*C*N), 96.4 (*s*, *C*≡C–C), 69.7 (*s*, *C*H–C≡C), 65.3 (*s*, *C*H Cp), 2.5–2.6 (*s*, C*H ^i^*Pr), 1.1 (*br s*, *C*H_3_^*i*^Pr), 8.4 (*s*, *C*H_2_ cyclo­prop­yl), −0.4 (*s*, *C*H cyclo­prop­yl).

**Synthesis of Yb[(CyN)_2_C—C**≡**C—Ph]_3_ (Cy = cyclo­hex­yl) (2)**

Anhydrous ytterbium(III) trichloride (1.40 g, 5.0 mmol) (Freeman & Smith, 1958 ▸) was suspended in THF (50 ml) and treated with a solution of Li[Ph—C≡C—C(NCy)_2_] (4.72 g, 15.0 mmol) (prepared *in situ* by addition of lithium phenyl­acetyl­ide to *N*,*N*′-di­cyclo­hexyl­carbodi­imide) in THF (60 ml). The reaction mixture was refluxed for 3 h. After cooling to room temperature, the white precipitate (LiCl) was removed by filtration, and the clear filtrate was evaporated to dryness. Off-white air- and moisture-sensitive solid. Yield: 3.07 g, 56%. M.p.: 505 K. Single crystal suitable for X-ray structure determination were obtained from a saturated toluene solution at 281 K. Analysis calculated for C_63_H_81_N_6_Yb: C 69.07, H 7.45, N 7.67%; found: C 69.21, H 7.50, N 7.47%. MS (EI, *M* = 1095.42): *m*/*z* (%) 1014 (23) [*M* – Cy]^+^, 1006 (7) [*M* – PhC]^+^, 998 (15), 964 (14), 949 (16), 899 (46), 849 (30), 833 (20), 811 (12), 799 (23), 787 (75) [*M* − NCy—C(C≡C—Ph)—NCy]^+^, 783 (35), 733 (62), 711 (6) [*M* − NCy—C(C≡C—Ph)-NCy – Ph]^+^, 683 (45), 667 (100) [*M* − NCy—C(C≡C—Ph)-NCy − Ph – C_3_H_8_]^+^, 645 (29). IR (KBr) ν (cm^−1^): 2922 (*s*), 2850 (*m*), 2661 (*w*), 2208 (*w*, C≡C), 1982 (*w*), 1598 (*w*), 1574 (*w*, NCN), 1491 (*m*), 1461 (*vs*), 1449 (*s*), 1411 (*m*), 1398 (*m*), 1343 (*s*), 1311 (*m*), 1256 (*m*), 1192 (*m*), 1170 (*m*), 1137 (*m*), 1070 (*m*), 1027 (*w*), 995 (*m*), 914 (*w*), 898 (*m*), 887 (*m*), 844 (*w*), 798 (*w*), 754 (*s*), 702 (*m*), 688 (*s*), 628 (*s*), 553 (*w*), 529 (*m*), 504 (*w*), 488 (*w*), 452 (*w*), 411 (*m*), 355 (*m*), 316 (*m*), 273 (*w*). ^1^H NMR (400.1 MHz, [D_8_]-THF, 298 K): 14.12 (*br s*, C*H*_2_, Cy), 6.88 (*br s*,C*H*_2_, Cy), 4.54 (*m*, 3H, *p*-C*H* Ph), 3.94 (*m*, 6H, *m*-C*H* Ph), 1.29 (*br s*, C*H*_2_, Cy), −0.15 (*d*, 6H, *o*-C*H* Ph), −14.62 (*br s*, N—C*H*, Cy). ^13^C NMR (100.6 MHz, [D_8_]-THF, 298 K): 126.4 (*s*, *p*-*C*H Ph), 126.0 (*s*, *m*-*C*H Ph), 124.6 (*s*, *o*-*C*H Ph), 111.2 (*s*, *i*-*C* Ph), 71.0 (*s*, ≡*C*-Ph), 46.0 (*s*, N-*C*H, Cy), 36.1 (*s*, *C*H_2_, Cy), 35.7 (*s*, *C*H_2_, Cy), 35.0 (*s*, *C*H_2_, Cy), ≡*C*-C(NCy)_2_ and N*C*N not observed.

## Refinement   

Crystal data, data collection and structure refinement details are summarized in Table 1 ▸. In the case of compound **2**, C atoms C17–C21 of the disordered cyclo­hexyl substituent have been split over two sites, with a freely refined occupancy ratio. The N-bonded C atom C16 was refined as not disordered using EXYZ and EADP commands but the different orientation of the corresponding H atom H17 was taken into account. The contribution to the scattering from the solvent molecule in compound **2** was removed with the SQUEEZE routine (Spek, 2015 ▸) in *PLATON* (Spek, 2009 ▸), yielding a solvent accessible volume of 1316 Å^3^ and 138 electrons. H atoms were fixed geometrically and refined using a riding model with *U*(H) = 1.20*U*_eq_(C).

## Supplementary Material

Crystal structure: contains datablock(s) li0090, li0065_sq. DOI: 10.1107/S2056989016012135/zl2671sup1.cif

Structure factors: contains datablock(s) li0090. DOI: 10.1107/S2056989016012135/zl2671li0090sup2.hkl

Structure factors: contains datablock(s) li0065_sq. DOI: 10.1107/S2056989016012135/zl2671li0065_sqsup4.hkl

CCDC references: 1492937, 1492936

Additional supporting information:  crystallographic information; 3D view; checkCIF report

## Figures and Tables

**Figure 2:**
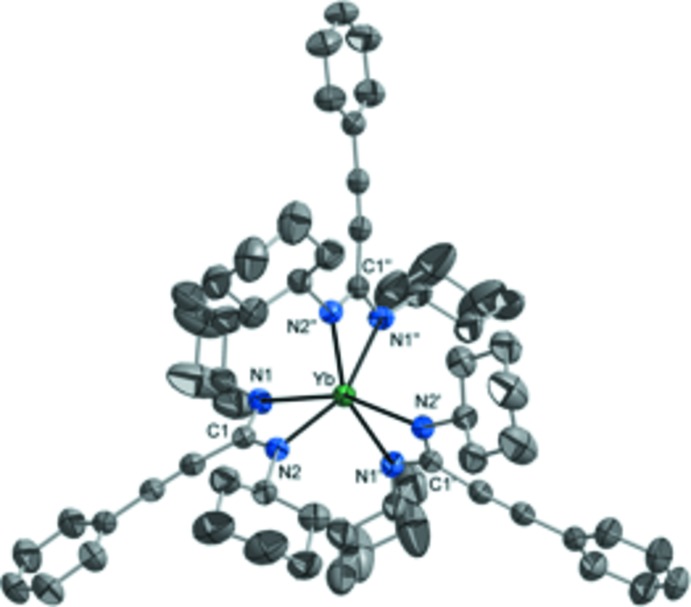
The mol­ecular structure of compound **2**. Displacement ellipsoids are at the 50% probability level and H atoms have been omitted for clarity. Only one orientation of the disordered cyclo­hexyl group at N2 is shown. The Yb atom is located on a threefold rotation axis parallel to the crystallographic *c* axis. [Symmetry operators to generate equivalent atoms: (′) 1 − *y*, −1 + *x* − *y*, *z*; (′′) 2 − *x* + *y*, 1 − *x*, *z*.]

**Table 1:** Experimental details

	**1**	**2**
Crystal data		
Chemical formula	[Yb(C_5_H_5_)_2_(C_12_H_19_N_2_)]	[Yb(C_21_H_27_N_2_)_3_]
*M*_r_	494.51	1095.37
Crystal system, space group	Orthorhombic, *P* *b* *c* *a*	Trigonal, *R* 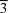 *c*:*H*
Temperature (K)	153	153
*a*, *b*, *c* (Å)	9.4578 (2), 19.2910 (6), 22.2114 (5)	20.3469 (3), 20.3469 (3), 50.3074 (11)
α, β, γ (°)	90, 90, 90	90, 90, 120
*V* (Å^3^)	4052.48 (18)	18036.8 (7)
*Z*	8	12
Radiation type	Mo *K*α	Mo *K*α
μ (mm^−1^)	4.62	1.60
Crystal size (mm)	0.33 × 0.31 × 0.25	0.36 × 0.35 × 0.24

Data collection		
Diffractometer	Stoe IPDS 2T	Stoe IPDS 2T
Absorption correction	Numerical (*X-AREA* and *X-RED*; Stoe & Cie, 2002 ▸)	Numerical (*X-AREA* and *X-RED*; Stoe & Cie, 2002 ▸)
*T*_min_, *T*_max_	0.326, 0.448	0.621, 0.722
No. of measured, independent and observed [*I* > 2σ(*I*)] reflections	21905, 4045, 3263	36832, 3578, 2774
*R*_int_	0.051	0.065
(sin θ/λ)_max_ (Å^−1^)	0.620	0.597

Refinement		
*R*[*F* ^2^ > 2σ(*F* ^2^)], *wR*(*F* ^2^), *S*	0.018, 0.040, 0.98	0.029, 0.071, 1.07
No. of reflections	4045	3578
No. of parameters	227	257
H-atom treatment	H-atom parameters constrained	H-atom parameters constrained
Δρ_max_, Δρ_min_ (e Å^−3^)	0.50, −0.72	0.31, −1.18

Computer programs: *X-AREA* and *X-RED* (Stoe & Cie, 2002 ▸), *SHELXS2013* (Sheldrick, 2008 ▸), *SHELXL2014* (Sheldrick, 2015 ▸) and *DIAMOND* (Brandenburg, 1999 ▸).

## supplementary crystallographic information

### (li0090) Bis(η5-cyclopentadienyl)(3-cyclopropyl-N,N'-diisopropylpropynamidinato-κ2N,N')ytterbium(III) Crystal data

**Table:**

[Yb(C_5_H_5_)_2_(C_12_H_19_N_2_)]	*D*_x_ = 1.621 Mg m^−^^3^
*M**_r_* = 494.51	Mo *K*α radiation, λ = 0.71073 Å
Orthorhombic, *P**b**c**a*	Cell parameters from 21907 reflections
*a* = 9.4578 (2) Å	θ = 1.8–26.2°
*b* = 19.2910 (6) Å	µ = 4.62 mm^−^^1^
*c* = 22.2114 (5) Å	*T* = 153 K
*V* = 4052.48 (18) Å^3^	Block, orange
*Z* = 8	0.33 × 0.31 × 0.25 mm
*F*(000) = 1960	

### (li0090) Bis(η5-cyclopentadienyl)(3-cyclopropyl-N,N'-diisopropylpropynamidinato-κ2N,N')ytterbium(III) Data collection

**Table:**

Stoe IPDS 2T diffractometer	4045 independent reflections
Radiation source: fine-focus sealed tube	3263 reflections with *I* > 2σ(*I*)
Detector resolution: 6.67 pixels mm^-1^	*R*_int_ = 0.051
area detector scans	θ_max_ = 26.2°, θ_min_ = 2.3°
Absorption correction: numerical (X-AREA and X-RED; Stoe & Cie, 2002)	*h* = −11→10
*T*_min_ = 0.326, *T*_max_ = 0.448	*k* = −23→22
21905 measured reflections	*l* = −27→27

### (li0090) Bis(η5-cyclopentadienyl)(3-cyclopropyl-N,N'-diisopropylpropynamidinato-κ2N,N')ytterbium(III) Refinement

**Table:**

Refinement on *F*^2^	Secondary atom site location: difference Fourier map
Least-squares matrix: full	Hydrogen site location: inferred from neighbouring sites
*R*[*F*^2^ > 2σ(*F*^2^)] = 0.018	H-atom parameters constrained
*wR*(*F*^2^) = 0.040	*w* = 1/[σ^2^(*F*_o_^2^) + (0.0189*P*)^2^] where *P* = (*F*_o_^2^ + 2*F*_c_^2^)/3
*S* = 0.98	(Δ/σ)_max_ < 0.001
4045 reflections	Δρ_max_ = 0.50 e Å^−^^3^
227 parameters	Δρ_min_ = −0.72 e Å^−^^3^
0 restraints	Extinction correction: SHELXL2014 (Sheldrick, 2015), Fc^*^=kFc[1+0.001xFc^2^λ^3^/sin(2θ)]^-1/4^
Primary atom site location: heavy-atom method	Extinction coefficient: 0.00042 (3)

### (li0090) Bis(η5-cyclopentadienyl)(3-cyclopropyl-N,N'-diisopropylpropynamidinato-κ2N,N')ytterbium(III) Special details

**Table:**

Geometry. All esds (except the esd in the dihedral angle between two l.s. planes) are estimated using the full covariance matrix. The cell esds are taken into account individually in the estimation of esds in distances, angles and torsion angles; correlations between esds in cell parameters are only used when they are defined by crystal symmetry. An approximate (isotropic) treatment of cell esds is used for estimating esds involving l.s. planes.

### (li0090) Bis(η5-cyclopentadienyl)(3-cyclopropyl-N,N'-diisopropylpropynamidinato-κ2N,N')ytterbium(III) Fractional atomic coordinates and isotropic or equivalent isotropic displacement parameters (Å^2^)

**Table:**

	*x*	*y*	*z*	*U*_iso_*/*U*_eq_	
C1	0.3923 (3)	0.02660 (12)	0.12656 (11)	0.0209 (5)	
C2	0.4476 (3)	−0.03844 (14)	0.14935 (12)	0.0243 (5)	
C3	0.5016 (3)	−0.08748 (13)	0.17234 (12)	0.0237 (5)	
C4	0.5728 (3)	−0.14441 (13)	0.20074 (11)	0.0239 (5)	
H1	0.5401	−0.1564	0.2423	0.029*	
C5	0.7285 (3)	−0.15415 (18)	0.18859 (17)	0.0451 (9)	
H3	0.7748	−0.1214	0.1605	0.054*	
H2	0.7888	−0.1699	0.2224	0.054*	
C6	0.6272 (4)	−0.20392 (15)	0.16337 (14)	0.0363 (7)	
H4	0.6240	−0.2509	0.1813	0.044*	
H5	0.6100	−0.2024	0.1194	0.044*	
C7	0.1682 (3)	0.00848 (14)	0.17826 (13)	0.0279 (6)	
H6	0.2233	−0.0186	0.2086	0.033*	
C8	0.0678 (3)	0.05675 (15)	0.21099 (14)	0.0354 (7)	
H9	0.0013	0.0294	0.2351	0.042*	
H7	0.1218	0.0876	0.2375	0.042*	
H8	0.0153	0.0845	0.1816	0.042*	
C9	0.0881 (3)	−0.04153 (15)	0.13814 (15)	0.0396 (7)	
H11	0.0232	−0.0693	0.1627	0.048*	
H10	0.0341	−0.0154	0.1081	0.048*	
H12	0.1553	−0.0722	0.1177	0.048*	
C10	0.6091 (3)	0.04894 (13)	0.07174 (12)	0.0265 (6)	
H13	0.6102	−0.0018	0.0627	0.032*	
C11	0.7191 (3)	0.06331 (19)	0.12009 (15)	0.0407 (8)	
H15	0.8127	0.0496	0.1054	0.049*	
H16	0.7193	0.1129	0.1297	0.049*	
H14	0.6961	0.0367	0.1564	0.049*	
C12	0.6454 (3)	0.08777 (16)	0.01454 (14)	0.0361 (7)	
H18	0.7412	0.0753	0.0017	0.043*	
H17	0.5779	0.0753	−0.0171	0.043*	
H19	0.6406	0.1378	0.0221	0.043*	
C13	0.3678 (4)	0.19289 (17)	0.20356 (15)	0.0468 (9)	
H20	0.3727	0.1576	0.2333	0.056*	
C14	0.4768 (4)	0.21487 (18)	0.16796 (18)	0.0513 (10)	
H21	0.5707	0.1974	0.1690	0.062*	
C15	0.4272 (4)	0.26772 (16)	0.12948 (17)	0.0458 (9)	
H22	0.4803	0.2920	0.0999	0.055*	
C16	0.2837 (3)	0.27732 (14)	0.14360 (13)	0.0333 (7)	
H23	0.2215	0.3098	0.1254	0.040*	
C17	0.2495 (4)	0.23090 (15)	0.18876 (14)	0.0368 (7)	
H24	0.1588	0.2260	0.2067	0.044*	
C18	0.2309 (3)	0.20903 (15)	−0.00641 (12)	0.0292 (6)	
H25	0.2880	0.2466	−0.0196	0.035*	
C19	0.1094 (3)	0.21427 (15)	0.02902 (12)	0.0308 (6)	
H26	0.0686	0.2558	0.0441	0.037*	
C20	0.0589 (3)	0.14683 (19)	0.03826 (14)	0.0456 (9)	
H27	−0.0230	0.1346	0.0606	0.055*	
C21	0.1492 (4)	0.10076 (17)	0.00905 (15)	0.0470 (9)	
H28	0.1402	0.0517	0.0086	0.056*	
C22	0.2543 (4)	0.13851 (15)	−0.01913 (13)	0.0368 (7)	
H29	0.3290	0.1201	−0.0429	0.044*	
N1	0.2658 (2)	0.05054 (11)	0.14281 (10)	0.0212 (4)	
N2	0.4664 (2)	0.06799 (10)	0.09026 (10)	0.0216 (4)	
Yb	0.29970 (2)	0.15407 (2)	0.09600 (2)	0.01886 (5)	

### (li0090) Bis(η5-cyclopentadienyl)(3-cyclopropyl-N,N'-diisopropylpropynamidinato-κ2N,N')ytterbium(III) Atomic displacement parameters (Å^2^)

**Table:**

	*U*^11^	*U*^22^	*U*^33^	*U*^12^	*U*^13^	*U*^23^
C1	0.0188 (12)	0.0168 (11)	0.0271 (13)	−0.0009 (10)	−0.0038 (11)	−0.0017 (9)
C2	0.0197 (13)	0.0225 (12)	0.0308 (13)	0.0006 (11)	0.0012 (10)	−0.0002 (11)
C3	0.0191 (13)	0.0243 (13)	0.0276 (14)	−0.0013 (12)	0.0011 (11)	−0.0005 (10)
C4	0.0233 (13)	0.0235 (13)	0.0248 (13)	0.0024 (11)	0.0008 (10)	0.0049 (10)
C5	0.0207 (16)	0.0513 (19)	0.063 (2)	0.0067 (15)	−0.0007 (14)	0.0297 (17)
C6	0.044 (2)	0.0315 (15)	0.0335 (16)	0.0146 (15)	0.0064 (14)	0.0086 (12)
C7	0.0194 (14)	0.0286 (14)	0.0356 (15)	0.0005 (11)	0.0026 (11)	0.0138 (12)
C8	0.0289 (15)	0.0425 (17)	0.0347 (15)	0.0003 (13)	0.0108 (13)	0.0069 (13)
C9	0.0248 (15)	0.0255 (14)	0.069 (2)	−0.0048 (14)	0.0090 (15)	0.0011 (14)
C10	0.0189 (13)	0.0207 (13)	0.0400 (14)	0.0017 (11)	0.0080 (12)	−0.0030 (11)
C11	0.0202 (16)	0.057 (2)	0.0453 (18)	0.0026 (14)	0.0030 (13)	0.0037 (15)
C12	0.0281 (15)	0.0424 (17)	0.0377 (17)	−0.0037 (14)	0.0093 (12)	−0.0038 (13)
C13	0.065 (2)	0.0368 (17)	0.0390 (18)	0.0037 (18)	−0.0226 (18)	−0.0134 (14)
C14	0.0307 (18)	0.0423 (19)	0.081 (3)	0.0127 (16)	−0.0246 (18)	−0.0393 (19)
C15	0.047 (2)	0.0275 (15)	0.063 (2)	−0.0175 (16)	0.0116 (17)	−0.0182 (15)
C16	0.0367 (18)	0.0198 (12)	0.0434 (16)	0.0058 (13)	−0.0073 (14)	−0.0059 (11)
C17	0.0409 (17)	0.0318 (15)	0.0377 (16)	−0.0032 (15)	0.0032 (14)	−0.0113 (12)
C18	0.0290 (17)	0.0302 (14)	0.0284 (14)	0.0040 (12)	−0.0004 (12)	0.0090 (11)
C19	0.0239 (14)	0.0404 (16)	0.0281 (14)	0.0095 (13)	−0.0019 (12)	0.0060 (12)
C20	0.0282 (16)	0.068 (2)	0.0405 (18)	−0.0194 (17)	−0.0139 (13)	0.0210 (17)
C21	0.069 (3)	0.0321 (16)	0.0396 (19)	−0.0118 (18)	−0.0301 (17)	0.0043 (13)
C22	0.0532 (19)	0.0357 (16)	0.0216 (14)	0.0131 (14)	−0.0055 (13)	0.0006 (11)
N1	0.0165 (11)	0.0190 (10)	0.0281 (11)	0.0019 (9)	0.0014 (9)	0.0040 (8)
N2	0.0169 (11)	0.0182 (10)	0.0298 (12)	0.0014 (8)	0.0024 (9)	−0.0018 (9)
Yb	0.01845 (6)	0.01490 (6)	0.02324 (6)	0.00096 (4)	−0.00219 (4)	0.00011 (4)

### (li0090) Bis(η5-cyclopentadienyl)(3-cyclopropyl-N,N'-diisopropylpropynamidinato-κ2N,N')ytterbium(III) Geometric parameters (Å, º)

**Table:**

C1—N1	1.332 (3)	C12—H19	0.9800
C1—N2	1.334 (3)	C13—C14	1.367 (6)
C1—C2	1.451 (4)	C13—C17	1.378 (5)
C1—Yb	2.697 (2)	C13—Yb	2.585 (3)
C2—C3	1.190 (4)	C13—H20	0.9500
C3—C4	1.434 (4)	C14—C15	1.411 (5)
C4—C6	1.507 (4)	C14—Yb	2.595 (3)
C4—C5	1.509 (4)	C14—H21	0.9500
C4—H1	1.0000	C15—C16	1.405 (5)
C5—C6	1.468 (5)	C15—Yb	2.610 (3)
C5—H3	0.9900	C15—H22	0.9500
C5—H2	0.9900	C16—C17	1.383 (4)
C6—H4	0.9900	C16—Yb	2.607 (3)
C6—H5	0.9900	C16—H23	0.9500
C7—N1	1.460 (3)	C17—Yb	2.582 (3)
C7—C9	1.516 (4)	C17—H24	0.9500
C7—C8	1.516 (4)	C18—C19	1.396 (4)
C7—H6	1.0000	C18—C22	1.407 (4)
C8—H9	0.9800	C18—Yb	2.592 (3)
C8—H7	0.9800	C18—H25	0.9500
C8—H8	0.9800	C19—C20	1.401 (4)
C9—H11	0.9800	C19—Yb	2.608 (3)
C9—H10	0.9800	C19—H26	0.9500
C9—H12	0.9800	C20—C21	1.393 (5)
C10—N2	1.458 (3)	C20—Yb	2.617 (3)
C10—C12	1.514 (4)	C20—H27	0.9500
C10—C11	1.521 (4)	C21—C22	1.382 (5)
C10—H13	1.0000	C21—Yb	2.610 (3)
C11—H15	0.9800	C21—H28	0.9500
C11—H16	0.9800	C22—Yb	2.610 (3)
C11—H14	0.9800	C22—H29	0.9500
C12—H18	0.9800	N1—Yb	2.274 (2)
C12—H17	0.9800	N2—Yb	2.293 (2)
			
N1—C1—N2	115.4 (2)	Yb—C18—H25	116.2
N1—C1—C2	122.0 (2)	C18—C19—C20	107.2 (3)
N2—C1—C2	122.6 (2)	C18—C19—Yb	73.82 (16)
N1—C1—Yb	57.36 (12)	C20—C19—Yb	74.83 (16)
N2—C1—Yb	58.18 (12)	C18—C19—H26	126.4
C2—C1—Yb	173.46 (18)	C20—C19—H26	126.4
C3—C2—C1	172.8 (3)	Yb—C19—H26	117.1
C2—C3—C4	177.1 (3)	C21—C20—C19	108.4 (3)
C3—C4—C6	120.1 (2)	C21—C20—Yb	74.25 (18)
C3—C4—C5	118.3 (2)	C19—C20—Yb	74.06 (16)
C6—C4—C5	58.2 (2)	C21—C20—H27	125.8
C3—C4—H1	116.0	C19—C20—H27	125.8
C6—C4—H1	116.0	Yb—C20—H27	117.8
C5—C4—H1	116.0	C22—C21—C20	108.4 (3)
C6—C5—C4	60.8 (2)	C22—C21—Yb	74.66 (18)
C6—C5—H3	117.7	C20—C21—Yb	74.84 (18)
C4—C5—H3	117.7	C22—C21—H28	125.8
C6—C5—H2	117.7	C20—C21—H28	125.8
C4—C5—H2	117.7	Yb—C21—H28	116.7
H3—C5—H2	114.8	C21—C22—C18	107.8 (3)
C5—C6—C4	61.0 (2)	C21—C22—Yb	74.64 (18)
C5—C6—H4	117.7	C18—C22—Yb	73.61 (16)
C4—C6—H4	117.7	C21—C22—H29	126.1
C5—C6—H5	117.7	C18—C22—H29	126.1
C4—C6—H5	117.7	Yb—C22—H29	117.7
H4—C6—H5	114.8	C1—N1—C7	121.4 (2)
N1—C7—C9	110.6 (2)	C1—N1—Yb	93.09 (15)
N1—C7—C8	108.3 (2)	C7—N1—Yb	145.49 (16)
C9—C7—C8	111.1 (2)	C1—N2—C10	120.4 (2)
N1—C7—H6	108.9	C1—N2—Yb	92.20 (15)
C9—C7—H6	108.9	C10—N2—Yb	146.57 (16)
C8—C7—H6	108.9	N1—Yb—N2	59.11 (7)
C7—C8—H9	109.5	N1—Yb—C17	96.51 (9)
C7—C8—H7	109.5	N2—Yb—C17	125.91 (9)
H9—C8—H7	109.5	N1—Yb—C13	82.36 (10)
C7—C8—H8	109.5	N2—Yb—C13	95.16 (10)
H9—C8—H8	109.5	C17—Yb—C13	30.92 (11)
H7—C8—H8	109.5	N1—Yb—C18	136.46 (9)
C7—C9—H11	109.5	N2—Yb—C18	114.82 (8)
C7—C9—H10	109.5	C17—Yb—C18	114.78 (10)
H11—C9—H10	109.5	C13—Yb—C18	139.01 (10)
C7—C9—H12	109.5	N1—Yb—C14	101.91 (11)
H11—C9—H12	109.5	N2—Yb—C14	85.28 (9)
H10—C9—H12	109.5	C17—Yb—C14	50.80 (11)
N2—C10—C12	108.8 (2)	C13—Yb—C14	30.60 (12)
N2—C10—C11	112.8 (2)	C18—Yb—C14	121.15 (12)
C12—C10—C11	110.3 (2)	N1—Yb—C16	127.40 (9)
N2—C10—H13	108.3	N2—Yb—C16	136.29 (9)
C12—C10—H13	108.3	C17—Yb—C16	30.92 (10)
C11—C10—H13	108.3	C13—Yb—C16	51.34 (10)
C10—C11—H15	109.5	C18—Yb—C16	88.18 (9)
C10—C11—H16	109.5	C14—Yb—C16	51.34 (10)
H15—C11—H16	109.5	N1—Yb—C19	123.68 (8)
C10—C11—H14	109.5	N2—Yb—C19	139.92 (8)
H15—C11—H14	109.5	C17—Yb—C19	94.16 (10)
H16—C11—H14	109.5	C13—Yb—C19	124.76 (10)
C10—C12—H18	109.5	C18—Yb—C19	31.15 (9)
C10—C12—H17	109.5	C14—Yb—C19	126.54 (11)
H18—C12—H17	109.5	C16—Yb—C19	77.60 (9)
C10—C12—H19	109.5	N1—Yb—C21	85.14 (9)
H18—C12—H19	109.5	N2—Yb—C21	92.78 (10)
H17—C12—H19	109.5	C17—Yb—C21	135.74 (12)
C14—C13—C17	108.0 (3)	C13—Yb—C21	159.02 (13)
C14—C13—Yb	75.12 (19)	C18—Yb—C21	51.33 (10)
C17—C13—Yb	74.42 (17)	C14—Yb—C21	170.27 (13)
C14—C13—H20	126.0	C16—Yb—C21	128.90 (10)
C17—C13—H20	126.0	C19—Yb—C21	51.47 (10)
Yb—C13—H20	116.6	N1—Yb—C15	132.26 (10)
C13—C14—C15	108.9 (3)	N2—Yb—C15	107.84 (10)
C13—C14—Yb	74.29 (19)	C17—Yb—C15	51.36 (10)
C15—C14—Yb	74.87 (18)	C13—Yb—C15	51.56 (12)
C13—C14—H21	125.5	C18—Yb—C15	91.28 (11)
C15—C14—H21	125.5	C14—Yb—C15	31.44 (12)
Yb—C14—H21	117.2	C16—Yb—C15	31.25 (10)
C16—C15—C14	106.3 (3)	C19—Yb—C15	96.16 (11)
C16—C15—Yb	74.22 (17)	C21—Yb—C15	142.52 (11)
C14—C15—Yb	73.69 (17)	N1—Yb—C22	108.89 (9)
C16—C15—H22	126.8	N2—Yb—C22	88.59 (9)
C14—C15—H22	126.8	C17—Yb—C22	144.82 (10)
Yb—C15—H22	117.4	C13—Yb—C22	168.41 (11)
C17—C16—C15	107.6 (3)	C18—Yb—C22	31.38 (9)
C17—C16—Yb	73.57 (16)	C14—Yb—C22	139.59 (13)
C15—C16—Yb	74.53 (16)	C16—Yb—C22	119.52 (9)
C17—C16—H23	126.2	C19—Yb—C22	51.59 (9)
C15—C16—H23	126.2	C21—Yb—C22	30.70 (11)
Yb—C16—H23	117.7	C15—Yb—C22	116.85 (11)
C13—C17—C16	109.1 (3)	N1—Yb—C20	93.12 (9)
C13—C17—Yb	74.66 (17)	N2—Yb—C20	122.16 (10)
C16—C17—Yb	75.52 (17)	C17—Yb—C20	105.16 (12)
C13—C17—H24	125.4	C13—Yb—C20	133.23 (12)
C16—C17—H24	125.4	C18—Yb—C20	51.22 (9)
Yb—C17—H24	116.3	C14—Yb—C20	152.55 (11)
C19—C18—C22	108.2 (3)	C16—Yb—C20	101.36 (11)
C19—C18—Yb	75.03 (15)	C19—Yb—C20	31.11 (10)
C22—C18—Yb	75.01 (16)	C21—Yb—C20	30.91 (11)
C19—C18—H25	125.9	C15—Yb—C20	125.90 (12)
C22—C18—H25	125.9	C22—Yb—C20	51.00 (11)
			
C3—C4—C5—C6	−109.6 (3)	C19—C20—C21—Yb	66.8 (2)
C3—C4—C6—C5	106.6 (3)	C20—C21—C22—C18	1.1 (3)
C17—C13—C14—C15	−0.3 (3)	Yb—C21—C22—C18	−66.7 (2)
Yb—C13—C14—C15	67.5 (2)	C20—C21—C22—Yb	67.8 (2)
C17—C13—C14—Yb	−67.7 (2)	C19—C18—C22—C21	−0.9 (3)
C13—C14—C15—C16	0.5 (3)	Yb—C18—C22—C21	67.4 (2)
Yb—C14—C15—C16	67.6 (2)	C19—C18—C22—Yb	−68.3 (2)
C13—C14—C15—Yb	−67.1 (2)	N2—C1—N1—C7	174.8 (2)
C14—C15—C16—C17	−0.6 (3)	C2—C1—N1—C7	−8.1 (4)
Yb—C15—C16—C17	66.7 (2)	Yb—C1—N1—C7	179.6 (3)
C14—C15—C16—Yb	−67.2 (2)	N2—C1—N1—Yb	−4.8 (2)
C14—C13—C17—C16	−0.1 (3)	C2—C1—N1—Yb	172.3 (2)
Yb—C13—C17—C16	−68.3 (2)	C9—C7—N1—C1	−81.7 (3)
C14—C13—C17—Yb	68.2 (2)	C8—C7—N1—C1	156.3 (2)
C15—C16—C17—C13	0.4 (3)	C9—C7—N1—Yb	97.6 (3)
Yb—C16—C17—C13	67.7 (2)	C8—C7—N1—Yb	−24.4 (4)
C15—C16—C17—Yb	−67.3 (2)	N1—C1—N2—C10	177.0 (2)
C22—C18—C19—C20	0.3 (3)	C2—C1—N2—C10	0.0 (4)
Yb—C18—C19—C20	−68.0 (2)	Yb—C1—N2—C10	172.3 (3)
C22—C18—C19—Yb	68.3 (2)	N1—C1—N2—Yb	4.7 (2)
C18—C19—C20—C21	0.4 (3)	C2—C1—N2—Yb	−172.3 (2)
Yb—C19—C20—C21	−66.9 (2)	C12—C10—N2—C1	158.5 (2)
C18—C19—C20—Yb	67.3 (2)	C11—C10—N2—C1	−78.8 (3)
C19—C20—C21—C22	−0.9 (4)	C12—C10—N2—Yb	−35.6 (4)
Yb—C20—C21—C22	−67.7 (2)	C11—C10—N2—Yb	87.2 (4)

### (li0065_sq) Tris(3-phenyl-N,N'-dicyclohexylpropynamidinato-κ2N,N')ytterbium(III) Crystal data

**Table:**

[Yb(C_21_H_27_N_2_)_3_]	*D*_x_ = 1.210 Mg m^−^^3^
*M**_r_* = 1095.37	Mo *K*α radiation, λ = 0.71073 Å
Trigonal, *R*3*c*:*H*	Cell parameters from 36835 reflections
*a* = 20.3469 (3) Å	θ = 2.0–25.1°
*c* = 50.3074 (11) Å	µ = 1.60 mm^−^^1^
*V* = 18036.8 (7) Å^3^	*T* = 153 K
*Z* = 12	Block, light yellow
*F*(000) = 6852	0.36 × 0.35 × 0.24 mm

### (li0065_sq) Tris(3-phenyl-N,N'-dicyclohexylpropynamidinato-κ2N,N')ytterbium(III) Data collection

**Table:**

Stoe IPDS 2T diffractometer	3578 independent reflections
Radiation source: fine-focus sealed tube	2774 reflections with *I* > 2σ(*I*)
Detector resolution: 6.67 pixels mm^-1^	*R*_int_ = 0.065
area detector scans	θ_max_ = 25.1°, θ_min_ = 2.0°
Absorption correction: numerical (X-AREA and X-RED; Stoe & Cie, 2002)	*h* = −24→24
*T*_min_ = 0.621, *T*_max_ = 0.722	*k* = −24→24
36832 measured reflections	*l* = −59→59

### (li0065_sq) Tris(3-phenyl-N,N'-dicyclohexylpropynamidinato-κ2N,N')ytterbium(III) Refinement

**Table:**

Refinement on *F*^2^	Primary atom site location: heavy-atom method
Least-squares matrix: full	Secondary atom site location: difference Fourier map
*R*[*F*^2^ > 2σ(*F*^2^)] = 0.029	Hydrogen site location: inferred from neighbouring sites
*wR*(*F*^2^) = 0.071	H-atom parameters constrained
*S* = 1.07	*w* = 1/[σ^2^(*F*_o_^2^) + (0.0274*P*)^2^ + 35.9511*P*] where *P* = (*F*_o_^2^ + 2*F*_c_^2^)/3
3578 reflections	(Δ/σ)_max_ = 0.001
257 parameters	Δρ_max_ = 0.31 e Å^−^^3^
0 restraints	Δρ_min_ = −1.18 e Å^−^^3^

### (li0065_sq) Tris(3-phenyl-N,N'-dicyclohexylpropynamidinato-κ2N,N')ytterbium(III) Special details

**Table:**

Geometry. All esds (except the esd in the dihedral angle between two l.s. planes) are estimated using the full covariance matrix. The cell esds are taken into account individually in the estimation of esds in distances, angles and torsion angles; correlations between esds in cell parameters are only used when they are defined by crystal symmetry. An approximate (isotropic) treatment of cell esds is used for estimating esds involving l.s. planes.
Refinement. PLATON SQUEEZE (Spek, 2015)

### (li0065_sq) Tris(3-phenyl-N,N'-dicyclohexylpropynamidinato-κ2N,N')ytterbium(III) Fractional atomic coordinates and isotropic or equivalent isotropic displacement parameters (Å^2^)

**Table:**

	*x*	*y*	*z*	*U*_iso_*/*U*_eq_	Occ. (<1)
C1	0.11736 (16)	0.97236 (16)	0.14621 (6)	0.0488 (7)	
C2	0.18462 (17)	0.96500 (16)	0.14454 (7)	0.0553 (8)	
C3	0.24269 (17)	0.96456 (16)	0.14323 (7)	0.0569 (8)	
C4	0.31432 (16)	0.96673 (17)	0.14298 (7)	0.0552 (8)	
C5	0.36272 (19)	0.9967 (2)	0.16454 (8)	0.0644 (9)	
H1	0.3477	1.0145	0.1795	0.077*	
C6	0.4328 (2)	1.0010 (2)	0.16442 (9)	0.0773 (11)	
H2	0.4658	1.0217	0.1792	0.093*	
C7	0.4543 (2)	0.9751 (2)	0.14279 (10)	0.0853 (13)	
H3	0.5017	0.9768	0.1429	0.102*	
C8	0.4074 (2)	0.9468 (2)	0.12089 (10)	0.0847 (13)	
H4	0.4233	0.9302	0.1058	0.102*	
C9	0.3374 (2)	0.9423 (2)	0.12084 (9)	0.0712 (10)	
H5	0.3052	0.9228	0.1058	0.085*	
C10	0.06136 (18)	0.8906 (2)	0.10821 (7)	0.0610 (9)	
H6	0.0980	0.8741	0.1138	0.073*	
C11	0.0855 (2)	0.9272 (3)	0.08192 (8)	0.0935 (14)	
H8	0.1378	0.9707	0.0831	0.112*	
H7	0.0518	0.9468	0.0764	0.112*	
C12	0.0825 (3)	0.8709 (3)	0.06098 (11)	0.134 (2)	
H10	0.0972	0.8960	0.0434	0.161*	
H9	0.1190	0.8540	0.0657	0.161*	
C13	0.0034 (3)	0.8030 (3)	0.05952 (11)	0.131 (2)	
H12	−0.0324	0.8195	0.0536	0.157*	
H11	0.0023	0.7663	0.0463	0.157*	
C14	−0.0206 (2)	0.7657 (3)	0.08578 (11)	0.1059 (18)	
H13	0.0125	0.7450	0.0910	0.127*	
H14	−0.0733	0.7227	0.0846	0.127*	
C15	−0.0164 (2)	0.82102 (19)	0.10691 (8)	0.0691 (10)	
H15	−0.0544	0.8365	0.1029	0.083*	
H16	−0.0290	0.7955	0.1244	0.083*	
C16A	0.17017 (18)	1.05213 (17)	0.18498 (7)	0.0566 (8)	0.442 (4)
H17A	0.2173	1.0728	0.1739	0.068*	0.442 (4)
C16B	0.17017 (18)	1.05213 (17)	0.18498 (7)	0.0566 (8)	0.558 (4)
H17B	0.1932	1.0199	0.1891	0.068*	0.558 (4)
C17A	0.1797 (4)	1.0071 (4)	0.20382 (16)	0.065 (2)	0.442 (4)
H18A	0.1894	0.9698	0.1946	0.078*	0.442 (4)
H19A	0.1324	0.9786	0.2142	0.078*	0.442 (4)
C17B	0.1324 (4)	1.0618 (3)	0.21256 (12)	0.0644 (17)	0.558 (4)
H18B	0.1064	1.0909	0.2087	0.077*	0.558 (4)
H19B	0.0938	1.0111	0.2191	0.077*	0.558 (4)
C18A	0.2455 (6)	1.0543 (5)	0.2227 (2)	0.091 (3)	0.442 (4)
H20A	0.2459	1.0217	0.2373	0.109*	0.442 (4)
H21A	0.2942	1.0753	0.2130	0.109*	0.442 (4)
C18B	0.1916 (6)	1.1025 (6)	0.23422 (18)	0.080 (3)	0.558 (4)
H20B	0.1677	1.1124	0.2497	0.096*	0.558 (4)
H21B	0.2121	1.0699	0.2402	0.096*	0.558 (4)
C19A	0.2374 (8)	1.1184 (9)	0.2342 (2)	0.089 (4)	0.442 (4)
H22A	0.2808	1.1492	0.2462	0.106*	0.442 (4)
H23A	0.1904	1.0971	0.2450	0.106*	0.442 (4)
C19B	0.2552 (6)	1.1765 (6)	0.2237 (2)	0.074 (3)	0.558 (4)
H22B	0.2949	1.2007	0.2375	0.088*	0.558 (4)
H23B	0.2354	1.2111	0.2198	0.088*	0.558 (4)
C20A	0.2345 (7)	1.1693 (8)	0.2125 (2)	0.067 (3)	0.442 (4)
H24A	0.2269	1.2094	0.2206	0.080*	0.442 (4)
H25A	0.2829	1.1940	0.2026	0.080*	0.442 (4)
C20B	0.2903 (3)	1.1658 (3)	0.19873 (14)	0.0672 (18)	0.558 (4)
H24B	0.3151	1.1359	0.2029	0.081*	0.558 (4)
H25B	0.3294	1.2159	0.1919	0.081*	0.558 (4)
C21A	0.1690 (4)	1.1208 (4)	0.19379 (14)	0.0533 (19)	0.442 (4)
H26A	0.1203	1.1055	0.2029	0.064*	0.442 (4)
H27A	0.1720	1.1514	0.1780	0.064*	0.442 (4)
C21B	0.2294 (3)	1.1248 (3)	0.17762 (11)	0.0516 (14)	0.558 (4)
H26B	0.2070	1.1568	0.1730	0.062*	0.558 (4)
H27B	0.2540	1.1196	0.1614	0.062*	0.558 (4)
N1	0.06247 (13)	0.94267 (14)	0.12836 (5)	0.0520 (6)	
N2	0.10931 (13)	1.01127 (13)	0.16578 (5)	0.0481 (6)	
Yb	0.0000	1.0000	0.14748 (2)	0.04418 (9)	

### (li0065_sq) Tris(3-phenyl-N,N'-dicyclohexylpropynamidinato-κ2N,N')ytterbium(III) Atomic displacement parameters (Å^2^)

**Table:**

	*U*^11^	*U*^22^	*U*^33^	*U*^12^	*U*^13^	*U*^23^
C1	0.0332 (15)	0.0310 (14)	0.080 (2)	0.0142 (12)	0.0002 (14)	0.0028 (14)
C2	0.0374 (16)	0.0342 (16)	0.091 (2)	0.0153 (13)	−0.0050 (16)	−0.0062 (15)
C3	0.0392 (17)	0.0312 (15)	0.096 (2)	0.0146 (13)	−0.0032 (16)	−0.0028 (15)
C4	0.0349 (15)	0.0358 (15)	0.094 (2)	0.0166 (13)	−0.0007 (16)	−0.0010 (16)
C5	0.0480 (18)	0.063 (2)	0.086 (2)	0.0308 (17)	−0.0031 (18)	−0.0010 (19)
C6	0.050 (2)	0.082 (3)	0.101 (3)	0.035 (2)	−0.013 (2)	−0.006 (2)
C7	0.044 (2)	0.081 (3)	0.139 (4)	0.037 (2)	−0.006 (2)	−0.009 (3)
C8	0.058 (2)	0.085 (3)	0.121 (4)	0.044 (2)	0.005 (2)	−0.019 (3)
C9	0.052 (2)	0.062 (2)	0.105 (3)	0.0325 (18)	−0.010 (2)	−0.015 (2)
C10	0.0388 (16)	0.062 (2)	0.083 (2)	0.0258 (15)	−0.0023 (16)	−0.0217 (18)
C11	0.070 (3)	0.095 (3)	0.086 (3)	0.018 (2)	0.015 (2)	−0.016 (2)
C12	0.084 (3)	0.155 (5)	0.106 (4)	0.017 (3)	0.026 (3)	−0.054 (4)
C13	0.060 (3)	0.167 (5)	0.121 (4)	0.024 (3)	0.004 (3)	−0.089 (4)
C14	0.058 (2)	0.089 (3)	0.163 (5)	0.031 (2)	−0.004 (3)	−0.065 (3)
C15	0.055 (2)	0.054 (2)	0.090 (3)	0.0210 (17)	0.0019 (19)	−0.0201 (19)
C16A	0.0441 (17)	0.0423 (17)	0.084 (2)	0.0221 (15)	−0.0159 (17)	−0.0112 (16)
C16B	0.0441 (17)	0.0423 (17)	0.084 (2)	0.0221 (15)	−0.0159 (17)	−0.0112 (16)
C17A	0.055 (5)	0.052 (4)	0.077 (5)	0.019 (4)	−0.015 (4)	0.006 (4)
C17B	0.054 (3)	0.044 (3)	0.070 (3)	0.006 (3)	0.007 (3)	−0.003 (3)
C18A	0.097 (7)	0.074 (6)	0.091 (7)	0.034 (6)	−0.042 (6)	0.006 (5)
C18B	0.085 (6)	0.058 (5)	0.068 (4)	0.014 (6)	−0.002 (5)	−0.003 (4)
C19A	0.091 (9)	0.088 (10)	0.066 (6)	0.029 (10)	−0.021 (7)	−0.011 (6)
C19B	0.066 (6)	0.051 (5)	0.078 (7)	0.010 (5)	−0.014 (5)	−0.010 (5)
C20A	0.065 (7)	0.058 (6)	0.067 (7)	0.024 (6)	−0.017 (5)	−0.015 (6)
C20B	0.043 (3)	0.045 (3)	0.099 (5)	0.012 (3)	−0.006 (3)	−0.003 (3)
C21A	0.053 (4)	0.055 (4)	0.048 (4)	0.023 (4)	−0.003 (3)	−0.006 (3)
C21B	0.044 (3)	0.035 (3)	0.067 (3)	0.014 (2)	0.002 (2)	0.003 (2)
N1	0.0380 (13)	0.0476 (14)	0.0731 (15)	0.0234 (11)	−0.0043 (13)	−0.0122 (13)
N2	0.0356 (13)	0.0367 (12)	0.0716 (16)	0.0177 (11)	−0.0075 (12)	−0.0059 (12)
Yb	0.03321 (10)	0.03321 (10)	0.06610 (15)	0.01661 (5)	0.000	0.000

### (li0065_sq) Tris(3-phenyl-N,N'-dicyclohexylpropynamidinato-κ2N,N')ytterbium(III) Geometric parameters (Å, º)

**Table:**

C1—N1	1.321 (4)	C16B—N2	1.459 (4)
C1—N2	1.324 (4)	C16B—C17B	1.645 (7)
C1—C2	1.451 (4)	C16B—H17B	1.0000
C1—Yb	2.714 (3)	C17A—C18A	1.528 (11)
C2—C3	1.188 (4)	C17A—H18A	0.9900
C3—C4	1.436 (4)	C17A—H19A	0.9900
C4—C5	1.385 (5)	C17B—C18B	1.526 (11)
C4—C9	1.393 (5)	C17B—H18B	0.9900
C5—C6	1.384 (5)	C17B—H19B	0.9900
C5—H1	0.9500	C18A—C19A	1.509 (18)
C6—C7	1.372 (6)	C18A—H20A	0.9900
C6—H2	0.9500	C18A—H21A	0.9900
C7—C8	1.381 (6)	C18B—C19B	1.508 (15)
C7—H3	0.9500	C18B—H20B	0.9900
C8—C9	1.382 (5)	C18B—H21B	0.9900
C8—H4	0.9500	C19A—C20A	1.53 (2)
C9—H5	0.9500	C19A—H22A	0.9900
C10—N1	1.458 (4)	C19A—H23A	0.9900
C10—C11	1.476 (6)	C19B—C20B	1.511 (13)
C10—C15	1.507 (4)	C19B—H22B	0.9900
C10—H6	1.0000	C19B—H23B	0.9900
C11—C12	1.536 (6)	C20A—C21A	1.525 (12)
C11—H8	0.9900	C20A—H24A	0.9900
C11—H7	0.9900	C20A—H25A	0.9900
C12—C13	1.510 (6)	C20B—C21B	1.524 (8)
C12—H10	0.9900	C20B—H24B	0.9900
C12—H9	0.9900	C20B—H25B	0.9900
C13—C14	1.480 (7)	C21A—H26A	0.9900
C13—H12	0.9900	C21A—H27A	0.9900
C13—H11	0.9900	C21B—H26B	0.9900
C14—C15	1.520 (5)	C21B—H27B	0.9900
C14—H13	0.9900	N1—Yb	2.320 (2)
C14—H14	0.9900	N2—Yb	2.310 (2)
C15—H15	0.9900	Yb—N2^i^	2.310 (2)
C15—H16	0.9900	Yb—N2^ii^	2.310 (2)
C16A—C17A	1.398 (8)	Yb—N1^i^	2.320 (2)
C16A—N2	1.459 (4)	Yb—N1^ii^	2.320 (2)
C16A—C21A	1.477 (8)	Yb—C1^ii^	2.714 (3)
C16A—H17A	1.0000	Yb—C1^i^	2.714 (3)
C16B—C21B	1.412 (6)		
			
N1—C1—N2	116.7 (3)	C19A—C18A—H21A	109.7
N1—C1—C2	122.6 (3)	C17A—C18A—H21A	109.7
N2—C1—C2	120.6 (3)	H20A—C18A—H21A	108.2
N1—C1—Yb	58.68 (15)	C19B—C18B—C17B	109.9 (7)
N2—C1—Yb	58.25 (15)	C19B—C18B—H20B	109.7
C2—C1—Yb	174.4 (2)	C17B—C18B—H20B	109.7
C3—C2—C1	175.2 (3)	C19B—C18B—H21B	109.7
C2—C3—C4	176.7 (4)	C17B—C18B—H21B	109.7
C5—C4—C9	119.4 (3)	H20B—C18B—H21B	108.2
C5—C4—C3	119.6 (3)	C18A—C19A—C20A	111.7 (10)
C9—C4—C3	120.9 (3)	C18A—C19A—H22A	109.3
C6—C5—C4	120.5 (4)	C20A—C19A—H22A	109.3
C6—C5—H1	119.8	C18A—C19A—H23A	109.3
C4—C5—H1	119.8	C20A—C19A—H23A	109.3
C7—C6—C5	119.7 (4)	H22A—C19A—H23A	107.9
C7—C6—H2	120.2	C18B—C19B—C20B	112.3 (8)
C5—C6—H2	120.2	C18B—C19B—H22B	109.1
C6—C7—C8	120.5 (4)	C20B—C19B—H22B	109.1
C6—C7—H3	119.7	C18B—C19B—H23B	109.1
C8—C7—H3	119.7	C20B—C19B—H23B	109.1
C9—C8—C7	120.2 (4)	H22B—C19B—H23B	107.9
C9—C8—H4	119.9	C21A—C20A—C19A	108.8 (10)
C7—C8—H4	119.9	C21A—C20A—H24A	109.9
C8—C9—C4	119.7 (4)	C19A—C20A—H24A	109.9
C8—C9—H5	120.2	C21A—C20A—H25A	109.9
C4—C9—H5	120.2	C19A—C20A—H25A	109.9
N1—C10—C11	112.1 (3)	H24A—C20A—H25A	108.3
N1—C10—C15	109.9 (3)	C19B—C20B—C21B	110.1 (6)
C11—C10—C15	111.3 (3)	C19B—C20B—H24B	109.6
N1—C10—H6	107.8	C21B—C20B—H24B	109.6
C11—C10—H6	107.8	C19B—C20B—H25B	109.6
C15—C10—H6	107.8	C21B—C20B—H25B	109.6
C10—C11—C12	111.1 (4)	H24B—C20B—H25B	108.1
C10—C11—H8	109.4	C16A—C21A—C20A	112.0 (7)
C12—C11—H8	109.4	C16A—C21A—H26A	109.2
C10—C11—H7	109.4	C20A—C21A—H26A	109.2
C12—C11—H7	109.4	C16A—C21A—H27A	109.2
H8—C11—H7	108.0	C20A—C21A—H27A	109.2
C13—C12—C11	109.9 (4)	H26A—C21A—H27A	107.9
C13—C12—H10	109.7	C16B—C21B—C20B	115.1 (5)
C11—C12—H10	109.7	C16B—C21B—H26B	108.5
C13—C12—H9	109.7	C20B—C21B—H26B	108.5
C11—C12—H9	109.7	C16B—C21B—H27B	108.5
H10—C12—H9	108.2	C20B—C21B—H27B	108.5
C14—C13—C12	110.7 (4)	H26B—C21B—H27B	107.5
C14—C13—H12	109.5	C1—N1—C10	120.5 (3)
C12—C13—H12	109.5	C1—N1—Yb	92.22 (18)
C14—C13—H11	109.5	C10—N1—Yb	147.1 (2)
C12—C13—H11	109.5	C1—N2—C16B	120.6 (2)
H12—C13—H11	108.1	C1—N2—C16A	120.6 (2)
C13—C14—C15	111.2 (4)	C1—N2—Yb	92.59 (18)
C13—C14—H13	109.4	C16B—N2—Yb	145.21 (19)
C15—C14—H13	109.4	C16A—N2—Yb	145.21 (19)
C13—C14—H14	109.4	N2^i^—Yb—N2^ii^	105.17 (7)
C15—C14—H14	109.4	N2^i^—Yb—N2	105.17 (7)
H13—C14—H14	108.0	N2^ii^—Yb—N2	105.17 (7)
C10—C15—C14	111.8 (3)	N2^i^—Yb—N1^i^	58.20 (9)
C10—C15—H15	109.3	N2^ii^—Yb—N1^i^	156.22 (8)
C14—C15—H15	109.3	N2—Yb—N1^i^	96.18 (9)
C10—C15—H16	109.3	N2^i^—Yb—N1^ii^	96.18 (9)
C14—C15—H16	109.3	N2^ii^—Yb—N1^ii^	58.19 (9)
H15—C15—H16	107.9	N2—Yb—N1^ii^	156.22 (8)
C17A—C16A—N2	115.6 (4)	N1^i^—Yb—N1^ii^	104.02 (8)
C17A—C16A—C21A	119.3 (5)	N2^i^—Yb—N1	156.22 (8)
N2—C16A—C21A	109.1 (3)	N2^ii^—Yb—N1	96.18 (8)
C17A—C16A—H17A	103.5	N2—Yb—N1	58.19 (9)
N2—C16A—H17A	103.5	N1^i^—Yb—N1	104.02 (8)
C21A—C16A—H17A	103.5	N1^ii^—Yb—N1	104.02 (8)
C21B—C16B—N2	117.4 (4)	N2^i^—Yb—C1^ii^	103.62 (8)
C21B—C16B—C17B	107.5 (4)	N2^ii^—Yb—C1^ii^	29.16 (9)
N2—C16B—C17B	108.2 (3)	N2—Yb—C1^ii^	131.74 (9)
C21B—C16B—H17B	107.8	N1^i^—Yb—C1^ii^	131.99 (9)
N2—C16B—H17B	107.8	N1^ii^—Yb—C1^ii^	29.10 (9)
C17B—C16B—H17B	107.8	N1—Yb—C1^ii^	100.12 (8)
C16A—C17A—C18A	112.0 (6)	N2^i^—Yb—C1^i^	29.16 (9)
C16A—C17A—H18A	109.2	N2^ii^—Yb—C1^i^	131.74 (9)
C18A—C17A—H18A	109.2	N2—Yb—C1^i^	103.62 (8)
C16A—C17A—H19A	109.2	N1^i^—Yb—C1^i^	29.10 (9)
C18A—C17A—H19A	109.2	N1^ii^—Yb—C1^i^	100.12 (8)
H18A—C17A—H19A	107.9	N1—Yb—C1^i^	131.99 (9)
C18B—C17B—C16B	112.2 (6)	C1^ii^—Yb—C1^i^	119.946 (6)
C18B—C17B—H18B	109.2	N2^i^—Yb—C1	131.74 (9)
C16B—C17B—H18B	109.2	N2^ii^—Yb—C1	103.62 (8)
C18B—C17B—H19B	109.2	N2—Yb—C1	29.16 (9)
C16B—C17B—H19B	109.2	N1^i^—Yb—C1	100.12 (8)
H18B—C17B—H19B	107.9	N1^ii^—Yb—C1	131.98 (9)
C19A—C18A—C17A	110.0 (8)	N1—Yb—C1	29.10 (9)
C19A—C18A—H20A	109.7	C1^ii^—Yb—C1	119.947 (6)
C17A—C18A—H20A	109.7	C1^i^—Yb—C1	119.945 (6)
			
C9—C4—C5—C6	−1.4 (5)	N2—C16B—C21B—C20B	178.3 (4)
C3—C4—C5—C6	−178.4 (3)	C17B—C16B—C21B—C20B	56.2 (6)
C4—C5—C6—C7	−0.2 (6)	C19B—C20B—C21B—C16B	−59.6 (8)
C5—C6—C7—C8	1.7 (7)	N2—C1—N1—C10	−171.7 (3)
C6—C7—C8—C9	−1.7 (7)	C2—C1—N1—C10	9.7 (4)
C7—C8—C9—C4	0.0 (6)	Yb—C1—N1—C10	−176.7 (3)
C5—C4—C9—C8	1.5 (6)	N2—C1—N1—Yb	4.9 (3)
C3—C4—C9—C8	178.5 (3)	C2—C1—N1—Yb	−173.6 (3)
N1—C10—C11—C12	−179.1 (3)	C11—C10—N1—C1	−104.2 (4)
C15—C10—C11—C12	−55.5 (5)	C15—C10—N1—C1	131.5 (3)
C10—C11—C12—C13	57.3 (7)	C11—C10—N1—Yb	81.9 (5)
C11—C12—C13—C14	−57.7 (7)	C15—C10—N1—Yb	−42.4 (6)
C12—C13—C14—C15	56.7 (6)	N1—C1—N2—C16B	−174.1 (3)
N1—C10—C15—C14	179.0 (4)	C2—C1—N2—C16B	4.5 (4)
C11—C10—C15—C14	54.2 (5)	Yb—C1—N2—C16B	−169.2 (3)
C13—C14—C15—C10	−54.7 (5)	N1—C1—N2—C16A	−174.1 (3)
N2—C16A—C17A—C18A	178.9 (6)	C2—C1—N2—C16A	4.5 (4)
C21A—C16A—C17A—C18A	−47.8 (9)	Yb—C1—N2—C16A	−169.2 (3)
C21B—C16B—C17B—C18B	−53.7 (7)	N1—C1—N2—Yb	−5.0 (3)
N2—C16B—C17B—C18B	178.6 (6)	C2—C1—N2—Yb	173.6 (3)
C16A—C17A—C18A—C19A	51.2 (11)	C21B—C16B—N2—C1	82.5 (4)
C16B—C17B—C18B—C19B	52.9 (11)	C17B—C16B—N2—C1	−155.7 (3)
C17A—C18A—C19A—C20A	−57.9 (14)	C21B—C16B—N2—Yb	−78.2 (5)
C17B—C18B—C19B—C20B	−55.0 (12)	C17B—C16B—N2—Yb	43.5 (5)
C18A—C19A—C20A—C21A	57.1 (14)	C17A—C16A—N2—C1	−74.5 (5)
C18B—C19B—C20B—C21B	55.8 (11)	C21A—C16A—N2—C1	147.7 (4)
C17A—C16A—C21A—C20A	47.8 (9)	C17A—C16A—N2—Yb	124.7 (5)
N2—C16A—C21A—C20A	−176.2 (7)	C21A—C16A—N2—Yb	−13.1 (6)
C19A—C20A—C21A—C16A	−49.3 (12)		

Symmetry codes: (i) −*y*+1, *x*−*y*+2, *z*; (ii) −*x*+*y*−1, −*x*+1, *z*.
